# Growth Inhibitory, Bactericidal, and Morphostructural Effects of Dehydrocostus Lactone from *Magnolia sieboldii* Leaves on Antibiotic-Susceptible and -Resistant Strains of *Helicobacter pylori*

**DOI:** 10.1371/journal.pone.0095530

**Published:** 2014-04-18

**Authors:** Hyun-Kyung Lee, Ha Eun Song, Haeng-Byung Lee, Cheol-Soo Kim, Mamoru Koketsu, Luong Thi My Ngan, Young-Joon Ahn

**Affiliations:** 1 Interdisciplinary Program in Agricultural Biotechnology, College of Agriculture and Life Sciences, Seoul National University, Seoul, South Korea; 2 Biomodulation Major, Department of Agricultural Biotechnology, Seoul National University, Seoul, South Korea; 3 Halla Botanical Garden, Jeju City, Jeju, South Korea; 4 Department of Chemistry and Biomolecular Science, Gifu University, Gifu, Japan; 5 Department of Plant Biotechnology and Biotransformation, Faculty of Biology, Ho Chi Minh City University of Science, Vietnam National University, Ho Chi Minh City, Vietnam; Indian Institute of Science, India

## Abstract

*Helicobacter pylori* is associated with various diseases of the upper gastrointestinal tract, such as gastric inflammation and duodenal and gastric ulcers. The aim of the study was to assess anti-*H. pylori* effects of the sesquiterpene lactone dehydrocostus lactone (DCL) from *Magnolia sieboldii* leaves, compared to commercial pure DCL, two previously known sesquiterpene lactones (costunolide and parthenolide), (–)-epigallocatechin gallate, and four antibiotics. The antibacterial activity of natural DCL toward antibiotic-susceptible *H. pylori* ATCC 700392 and *H. pylori* ATCC 700824 strains (MIC, 4.9 and 4.4 mg/L) was similar to that of commercial DCL and was more effective than costunolide, parthenolide, and EGCG. The activity of DCL was slightly lower than that of metronidazole (MIC, 1.10 and 1.07 mg/L). The antibacterial activity of DCL was virtually identical toward susceptible and resistant strains, even though resistance to amoxicillin (MIC, 11.1 mg/L for PED 503G strain), clarithromycin (49.8 mg/L for PED 3582GA strain), metronidazole (21.6 mg/L for *H. pylori* ATCC 43504 strain; 71.1 mg/L for 221 strain), or tetracycline (14.2 mg/L for B strain) was observed. This finding indicates that DCL and the antibiotics do not share a common mode of action. The bactericidal activity of DCL toward *H. pylori* ATCC 43504 was not affected by pH values examined (4.0–7.0). DCL caused considerable conversion to coccoid form (94 versus 49% at 8 and 4 mg/L of DCL for 48 h). The Western blot analysis revealed that urease subunits (UreA and UreB) of *H. pylori* ATCC 43504 were not affected by 10 mM of DCL, whereas UreA monomer band completely disappeared at 0.1 mM of (–)-epigallocatechin gallate. Global efforts to reduce the level of antibiotics justify further studies on *M. sieboldii* leaf-derived materials containing DCL as potential antibacterial products or a lead molecule for the prevention or eradication of drug-resistant *H. pylori*.

## Introduction

*Helicobacter pylori* is strongly associated with a number of the most important diseases of the upper gastrointestinal tract, such as gastric inflammation, chronic superficial gastritis, duodenal and gastric ulcers, gastric adenocarcinoma, and non-Hodgkin's lymphomas of the human stomach [1], [2]. Infections are prevalent worldwide and especially more common among children in both developed and developing countries. In developing countries, 70–90% of population carries *H*. *pylori*, while the prevalence of infection in developed countries is lower, ranging from 25 to 50% [3], [4]. Triple therapies containing a proton pump inhibitor such as omeprazole and clarithromycin plus amoxicillin, metronidazole, or a fluoroquinolone are extremely sensitive to resistance to the third drug [5]–[8]. The recommended regimens for *H. pylori* therapy have been well described by Rimbara et al. [5] and Graham et al. [8]. For example, the bismuth quadruple therapy and nonbismuth concomitant quadruple therapy provide good results. Triple therapy causes mild but relatively frequent side effects such as taste disturbances, nausea, diarrhea, dyspepsia, headache, and angioedema [1], as well as disturbance of human gastrointestinal microflora [9], [10]. The cost of combination therapy is significant. In addition, a commercial vaccine is still not available. These problems highlight a critical need for the development of selective antibacterial agents with novel target sites to establish an effective drug-resistance management strategy and tactics based on all available information on the extent and nature of resistance in *H*. *pylori*.

Plant secondary metabolites have been suggested as potential alternatives for *H*. *pylori* therapy largely because plants constitute a potential source of bioactive chemicals that have been perceived by the general public as relatively safe and often act at multiple and novel target sites, thereby reducing the potential for resistance [11]. In addition, certain plant preparations and their constituents are highly effective toward drug-resistant strains of *H. pylori* [12], [13]. Much effort has been focused on them as potential sources of commercial antibacterial products for prevention or eradication of *H*. *pylori*. In particular, it was initially reported that a methanol extract from the leaves of *Magnolia sieboldii* K. Koch (Magnoliaceae) had good growth inhibitory activity toward *H*. *pylori* ATCC 43504 [14]. No information has been done to consider potential use of *M. sieboldii* to manage drug-resistant *H*. *pylori*, although pharmacological actions of the genus *Magnolia* have been well described by Lee et al. [15].

The aim of the study was to assess antibacterial effects on two antibiotic-susceptible strains and five antibiotic-resistant strains of *H. pylori* of the sesquiterpene lactone dehydrocostus lactone (DCL) from *Magnolia sieboldii* leaves, compared to commercial pure DCL, two previously known sesquiterpene lactones (costunolide and parthenolide), (–)-epigallocatechin gallate (EGCG), and four antibiotics.

## Materials and Methods

### Instrumental Analysis

^1^H and ^13^C NMR spectra were recorded in CDCl_3_ on a Bruker AM-500 spectrometer (Rheinstetten, Baden-Württemberg, Germany) using tetramethylsilane as an internal standard, and chemical shifts are given in δ (ppm). Distortionless enhancement by polarization transfer (DEPT) spectra was acquired using the Bruker software. UV spectra were obtained in methanol on a Jasco V-550 UV/VIS spectrophotometer (Tokyo, Japan), FT-IR spectra on a Midac Nicolet Magna 550 series II spectrometer (Irvine, CA), and mass spectra on a Jeol GSX 400 spectrometer (Tokyo, Japan). Optical rotation was measured with a Rudolph Research Analytical Autopol III polarimeter (Flanders, NJ). Merck silica gel (0.063–0.2 mm) (Darmstadt, Hesse, Germany) was used for column chromatography. Merck precoated silica gel plates (Kieselgel 60 F_254_) were used for analytical thin layer chromatography (TLC). A Thermo Separation Products Spectra System P2000 high-performance liquid chromatograph (HPLC) (San Jose, CA) was used for isolation of active principles.

### Plant Sample

The fresh leaves of *M. sieboldii* were collected from the Halla Botanical Garden (Jeju, Jeju Province, South Korea) in mid-July 2009. A certified botanical taxonomist was used to identify the plant. A voucher specimen (JI-70) was deposited in the Halla Botanical Garden and the Research Institute for Agriculture and Life Science, Seoul National University.

### Materials

**Figure 1:**
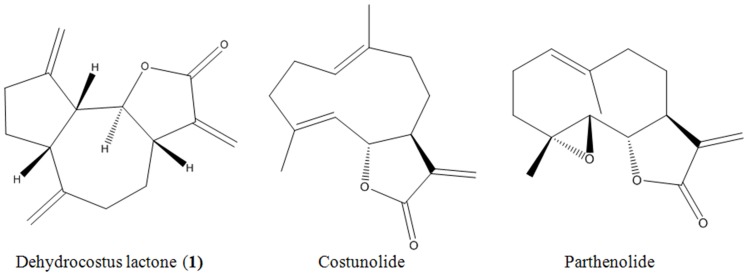
Structures of dehydrocostus lactone (1), costunolide, and parthenolide. Dehydrocostus lactone is the guaianolide sesquiterpenoid identified in *M*. *sieboldii* leaves in this study, and costunolide and parthenolide are previously known germacranolide sesquiterpenoids.

Pure organic DCL (≥98% purity), costunolide (≥97%) and parthenolide (≥98%) (Figure 1) and EGCG (≥95%) were purchased from Sigma-Aldrich (St. Louis, MO). Four antibiotics amoxicillin (≥97.0% purity), clarithromycin (≥98%), metronidazole (99%), and tetracycline (≥98.0%) were purchased from Sigma-Aldrich. Brucella broth and newborn bovine serum (NBS) were purchased from Becton, Dickinson and Company (Sparks, MD) and Hyclone (Longan, UT), respectively. A Bradford protein assay kit was purchased from Sigma-Aldrich. The protein molecular weight standards (Precision Plus Protein all blue standards) were supplied by Bio-Rad Life Sciences (Hercules, CA). All of the other chemicals and reagents used in this study were of analytical grade quality and available commercially.

### Bacterial Strains and Culture Conditions

The three reference strains (*H. pylori* ATCC 700392, *H. pylori* ATCC 700824, and *H. pylori* ATCC 43504) were purchased from American Type Culture Collection (ATCC) (Manassas, VA). Four clinical isolates (PED503G, PED 3582GA, 221, and B) of *H. pylori* were obtained in 2011 from the Culture Collection of Antimicrobial Resistant Microbes (Seoul, South Korea) from individual patients with gastric or duodenal ulcers. The bacterial strains were grown on Brucella agar supplemented with 10% NBS at 37°C for 72 h in an atmosphere of 5% O_2_, 15% CO_2_, and 80% N_2_ in an anaerobic chamber (Hirayama, Tokyo).

### Bioassay-Guided Fractionation and Isolation of Active Principles

The air-dried leaves (600 g) of *M*. *sieboldii* were pulverized, extracted with methanol (2×4 L) at room temperature, and filtered. To yield ∼31% as a dark greenish tar (based on the weight of the dried leaves), the combined filtrate was concentrated to dryness by rotary evaporation at 40°C. The extract (40 g) was sequentially partitioned into hexane- (9.6 g), chloroform- (1.6 g), ethyl acetate- (2.4 g), butanol- (9.0 g), and water-soluble (17.4 g) portions for subsequent bioassay. The organic solvent-soluble portions were concentrated under vacuum at 40°C and the water-soluble portion was freeze-dried. For isolation of active principles, 0.1 and 1 mg/paper disk (1 mm thickness, 8 mm diameter) of each *M*. *sieboldii* leaf-derived material toward *H*. *pylori* ATCC 43504 was tested in a paper-disk diffusion bioassay as described previously [16].

The hexane-soluble fraction (7 g) was most biologically active (Table 1) and was chromatographed on a 5.5×70 cm silica gel (600 g) column by elution with a gradient of hexane and ethyl acetate (100:0, 90:10, 80:20, 70:30, 60:40, 50:50, 20:80, and 0:100 by volume) and finally with methanol. Column fractions were monitored by TLC on silica gel plates developed with hexane and ethyl acetate (1:2 by volume) mobile phase. Fractions with similar *R_f_* values on the TLC plates were pooled. Spots were detected by spraying with 30% sulfuric acid and then heating on a hot plate. Eight fractions were obtained and bioassyed. A HPLC was used for further separation of the constituents from the active H3 fraction (4.99 g). The column was a 7.8 mm i.d. ×300 mm Waters μPorasil (Milford, CA) with a mobile phase of hexane and ethyl acetate (85:15 by volume) at a flow rate of 1 mL/min. Chromatographic separations were monitored using a UV detector at 265 nm. Finally, an active principle (730 mg) was isolated at a retention time of 23.3 min. The isolate was obtained as colorless powder and identified by instrumental analyses. The EI-MS revealed a molecular ion at m/z 230 [M]^+^ and its ^13^C NMR spectra showed 15 carbons in the molecule comprising seven methylenes, four methines, and four nonprotonated carbons as indicated in DEPT, suggesting the molecular formula C_15_H_18_O_2_. IR spectra of the isolate exhibited the presence of γ-lactone (1758 cm^−1^) and vinylidene group (3125, 1632, and 894 cm^−1^). This compound (**1**) was thus identified as dehydrocostus lactone (Figure 1). The interpretations of proton and carbon signals were largely consistent with those of Taniguchi et al. [17]. Dehydrocostus lactone was identified on the basis of the following evidence: colorless powder. [α]_D_^25^: −25° (*c* 0.008, CHCl_3_). UV (hexane) λ_max_ nm (ε): 220 (169.6). FT-IR *v*_max_ cm^−1^: 3125, 1758, 1632, 894. EI-MS (70 eV) *m/z* (% relative intensity): 230 [M]^+^ (62), 202 (13), 201 (18), 173 (13), 159 (14), 150 (39), 131 (23), 105 (43), 91 (96), 79 (83), 53 (100). ^1^H NMR (CDCl_3_, 400 MHz): δ 1.42 (1H, m), 1.91 (2H, m), 2.17 (1H, m), 2.25 (1H, m), 2.49 (1H, m), 2.50 (2H, m), 2.85 (1H, m), 2.89 (1H, m), 2.95 (1H, m), 3.95 (1H, dd, *J* = 8.8, 11.0 Hz), 4.81 (1H, s), 4.89 (1H, s), 5.07 (1H, d, *J* = 1.7 Hz), 5.27 (1H, d, *J* = 1.8 Hz), 5.49 (1H, d, *J* = 3.4 Hz), 6.21 (1H, d, *J* = 3.6 Hz). ^13^C NMR (CDCl_3_, 100 MHz): δ 30.2 t, 30.9 t, 32.6 t, 36.2 t, 45.1 d, 47.6 d, 52.0 d, 85.2 d, 109.6 t, 112.6 t, 120.1 t, 139.2 s, 149.2 s, 151.2 s, 170.2 s.

**Table 1:** Growth inhibitory activity of fractions obtained from the solvent hydrolyzable of the methanol extract of *M. sieboldii* leaves toward *H. pylori* ATCC 43504 using paper-disk diffusion bioassay.

Material	Inhibition zone (mm)	
	1 mg/disk	0.1 mg/disk
Hexane-soluble fraction	35	8
Chloroform-soluble fraction	28	8
Ethyl acetate-soluble fraction	9	8
Butanol-soluble fraction	8	8
Water-soluble fraction	8	8

### Microbiological Assay

A broth dilution assay in sterile 96-well plates and 15 mL tubes was used to evaluate the minimal inhibitory concentrations (MICs) [18] and minimal bactericidal concentrations (MBCs) [19] of the test compounds toward all strains of *H. pylori*, respectively. For MICs, 75 µL of the bacterial suspensions (5×10^6^ colony-forming unit (CFU)/mL) from the 72 h cultures on Brucella agar were inoculated with 75 µL of 10% NBS-supplemented Brucella broth containing serial twofold dilutions of each compound in dimethylsulfoxide (DMSO), and the plates were incubated at 37°C under microaerophilic conditions and shaken at 50 rpm for 48 h. The final concentration of DMSO in all assays was 2.5% or less. MICs were defined as the lowest concentrations that visibly inhibited bacterial growth using resazurin as an indicator. For MBCs and time killing assay, the 200 µL bacterial suspensions (1×10^7^ CFU/mL) were inoculated into 1.8 mL of 10% NBS-supplemented Brucella broth alone or containing various concentrations (0, 1, 1.5, or 2 times the MIC) of each compound and incubated with shaking (150 rpm) at 37°C for 48 h. Samples for viability measurement were taken after 0, 6, 12, 18, 24, 36, and 48 h, and 0.1 mL of 10-fold serial dilutions was plated onto Brucella agar without the test samples. Colonies were counted after 72 h incubation. Four antibiotics (amoxcillin, clarithromycin, metronidazole, and tetracycline), pure DCL, and EGCG served as positive controls and were similarly formulated. Negative controls consisted of the DMSO solution only. All bioassays were repeated three times in triplicate.

### Measurement of Bactericidal Activity at Various pHs

Because the prevalence of metronidazole resistance in many developing countries is greater than 40% (often 80% or greater) [8], the methods of Ohno et al. [20] and Shibata et al. [21] were used with a slight modification to assess the effects of pH on the bactericidal activity of DCL toward the metronidazole-resistant *H. pylori* ATCC43504. The buffer solutions used were 100 mM citrate buffer (pHs 4.0 and 5.0) and 10 mM sodium phosphate buffer (pHs 6.0 and 7.0). The 200 µL bacterial suspension of *H. pylori* ATCC 43504 (1×10^7^ CFU/mL) was added to 1.8 mL of each buffer containing DCL (0, 1, or 2 times the MIC). The cultures were incubated with shaking (150 rpm) at 37°C. Samples (0.1 mL) were taken at 0, 15, 30, and 60 min and plated onto fresh Brucella agar. Colonies were counted as stated previously. All bioassays were repeated three times in triplicates.

### Scanning Electron Microscopic Analysis

The bacterial pellets from cultures of *H. pylori* ATCC 700824 and five antibiotic-resistant strains of *H. pylori* after 48 h with or without DCL treatment at MIC concentrations were harvested by centrifugation at 800×*g* at 4°C for 5 min according to a modified method of Watt [22]. In brief, the specimens were fixed in modified Karnovsky's fixative (2% glutaraldehyde (v/v) and 2% paraformaldehyde (v/v) in 0.05 M sodium cacodylate buffer (pH 7.2)) [23] and post-fixed in 1% osmium tetroxide in the same buffer at 4°C for 2 h. Fixed samples were washed two times with the same buffer and distilled water. The samples were dehydrated through a series of increasing concentrations of ethanol up to 100%, and then treated with hexamethyldisilazane for 15 min (repeated twice), and dried at room temperature overnight. Specimens were then mounted on scanning electron microscope stubs by double-sided carbon conductive tape and coated with platinum (10 nm thickness) prior to visualization using a Carl Zeiss Supra-55 VP field emission scanning electron microscope (Jena, Thuringia, Germany).

### Microscopic Observation

*H. pylori* ATCC43504 was cultured in Brucella broth with or without DCL (4 and 8 mg/L) in microaerophilic conditions for 24 and 48 h. The 15 µL bacterial suspension was evenly spreaded and fixed on slides, and then stained with 0.3% methylene blue. Proportion of coccoid versus spiral bacteria was determined using a Carl Zeiss microscope equipped with AxioCam HRC camera. Counts of 200 bacteria from each slide were performed as reported previously by Cole et al. [24].

### Urease Inhibition by Indophenol Method

Urease crude of *H. pylori* ATCC 43504 was prepared as reported previously by Icatlo et al. [25]. The assay mixtures, containing 0.25 µg urease crude (0.04 urease units) in 100 µL of the EDTA-sodium phosphate buffer (pH 7.3) and each test compound (0.01–10 mM), were preincubated at 37°C for 2 h at 50 rpm. Inhibition of urease activity was determined using the indophenol method [26]. The ammonia released by the urease was quantified by measuring absorbance on a Molecular Devices Versa Max microplate reader (Sunnyvale, CA) at 625 nm with ammonium chloride as a standard. The protein content was determined using a Bradford protein assay kit. Bovine serum albumin was used as a protein standard. Because of its strong urease inhibitory activity of *H. pylori* [27], EGCG served as a reference standard and was similarly formulated. All bioassays were repeated three times in triplicate.

### Western Blotting

The mixtures of 50 µg urease crude (0.08 urease units) in 100 µL EDTA-sodium phosphate buffer and DCL (0.1–10 mM) were preincubated at 37°C for 1 h. The samples were mixed with 5× sample buffer containing sodium dodecyl sulfate (SDS), heated in 10 min, and then loaded onto SDS-PAGE polyacrylamide gels 12% (w/v). After electrophoresis at 120 V for 2 h, proteins from gels were transferred onto a Pall Corporation polyvinyl difluoride membrane (Pensacola, FL) [28]. The membrane was blocked with phosphate-buffered saline (PBS) containing 5% (v/v) skimmed milk for 2 h at room temperature. The membrane was then incubated overnight at 4°C with 1∶2000 dilution of Abcam duck polyclonal to *H. pylori* urease (Cambridge, MA). After being washed with PBS three times, the membrane was further incubated for 2 h with Abcam rabbit polyclonal secondary antibody to Abcam chicken IgY-H&L diluted 1∶4000. Finally, after several washings with PBS containing 0.5% Tween-20 (v/v), the blots were developed using an Amersham Biosciences ECL Western blotting detection reagent (Buckinghamshire, UK) and immediately exposed to an AGFA CP-PU X-ray film (Mortsel, Antwerp, Belgium) for 2–10 min at room temperature. Western blot results were analyzed using a Bio-Rad Molecular Imager Gel Doc XR system (Hercules, CA). EGCG served as a reference standard and was similarly prepared.

### Data Analysis

Percent urease inhibition was determined as reported previously [29]. Concentrations of the test compounds causing 50% loss of the urease activity (IC_50_) were determined using GraphPad Prism 5 software program (La Jolla, CA). Percentages of conversion to the coccoid form were transformed to arcsine square root values for analysis of variance. The Bonferroni multiple-comparison method was used to test for significant differences among the treatments (GraphPad Prism 5 software program). Resistance to amoxicillin, clarithromycin, metronidazole, and tetracycline was defined by MIC ≥1 mg/L, MIC ≥1 mg/L, MIC ≥8 mg/L, and MIC ≥4 mg/L, respectively, as described previously by Mégraud and Lehours [30].

## Results

### Growth Inhibitory Activity of Test Compounds

The growth inhibitory activities of three sesquiterpene lactones, EGCG, and four commercial antibiotics toward three reference strains and four clinical isolates of *H. pylori* were evaluated using a broth dilution assay (Table 2). Based on MIC values, the growth inhibitory effect of natural DCL was similar to that of commercial DCL, indicating that the activity of the methanol-extracted DCL is purely due to DCL. The constituent was more effective at inhibiting bacterial growth than costunolide, parthenolide, and EGCG. As for antibiotics, 23/28 MIC (82%) were better for antibiotics over DCL, with the exception of two metronidazole-resistant strains (*H. pylori* ATCC 43504 and 221), one amoxicillin-resistant strain (PED 503G), one clarithromycin-resistant strain (PED 3582GA), and one tetracycline-resistant strain (B).

**Table 2:** *In vitro* minimal inhibitory concentrations (MICs) and bactericidal concentrations (MBCs) of three sesquiterpene lactones, (–)-epigallocatechin gallate, and four commercial antibiotics toward three reference strains and four clinical isolates of *H. pylori* using broth dilution bioassay.

Compound^a^	ATCC 700392	ATCC 700824	ATCC 43504	PED 503G	PED 3582GA	221	B
	MIC^b^ (MBC^b^)	MIC (MBC)	MIC (MBC)	MIC (MBC)	MIC (MBC)	MIC (MBC)	MIC (MBC)
Natural DCL	4.9	4.4	4.0	4.9	6.7	4.9	6.7
	(7.6)	(10.7)	(7.6)	(7.6)	(9.8)	(8.9)	(10.1)
Pure DCL	4.9	4.4	4.0	4.9	6.7	4.9	6.7
	(7.6)	(10.7)	(7.6)	(7.6)	(9.8)	(8.9)	(10.1)
Costunolide	26.7	16.9	23.1	28.4	32	24.9	21.3
	(256)	(256)	(256)	(256)	(256)	(256)	(256)
Parthenolide	99.6	74.7	85.3	106.7	120.9	92.4	113.8
	(>2500)	(>2500)	(>2500)	(>2500)	(>2500)	(>2500)	(>2500)
EGCG	133.3	142.2	106.7	160	160	124.4	133.3
	(640)	(640)	(640)	(640)	(640)	(640)	(640)
Amoxicillin	0.024	0.012	0.029	**11.1**	0.44	0.062	2.44
	(0.062)	(0.031)	(0.067)	(15.1)	(0.98)	(0.098)	(3.11)
Clarithromycin	0.062	0.036	0.06	0.58	**49.8**	0.62	0.049
	(0.088)	(0.062)	(0.3)	(0.98)	(85.3)	(0.9)	(0.062)
Tetracycline	0.27	0.08	0.29	0.053	4.89	2.0	**14.2**
	(0.62)	(0.11)	(1.1)	(0.12)	(6.67)	(3.56)	(19.6)
Metronidazole	1.10	1.07	**21.6**	1.78	1.33	**71.1**	1.16
	(2.13)	(6.22)	(151.1)	(3.33)	(2.44)	(113.8)	(1.96)

a Natural DCL, dehydrocostus lactone isolated in this study; Pure DCL, commercially available dehydrocostus lactone; EGCG, (–)-epigallocatechin gallate.

b Unit, mg/L.

### Bactericidal Activity of Test Compounds

The MBCs of all compounds toward the seven strains examined are recorded in Table 2. As judged by MBC values, the bactericidal activity of natural DCL was similar to that of commercial DCL and was more effective than costunolide, parthenolide, and EGCG. Interestingly, all of the compounds were of nearly similar bactericidal activity toward both antibiotic-susceptible and -resistant strains, indicating a lack of resistance in the *H. pylori* ATCC 43504, PED503G, PED 3582GA, 221, and B.

**Figure 2:**
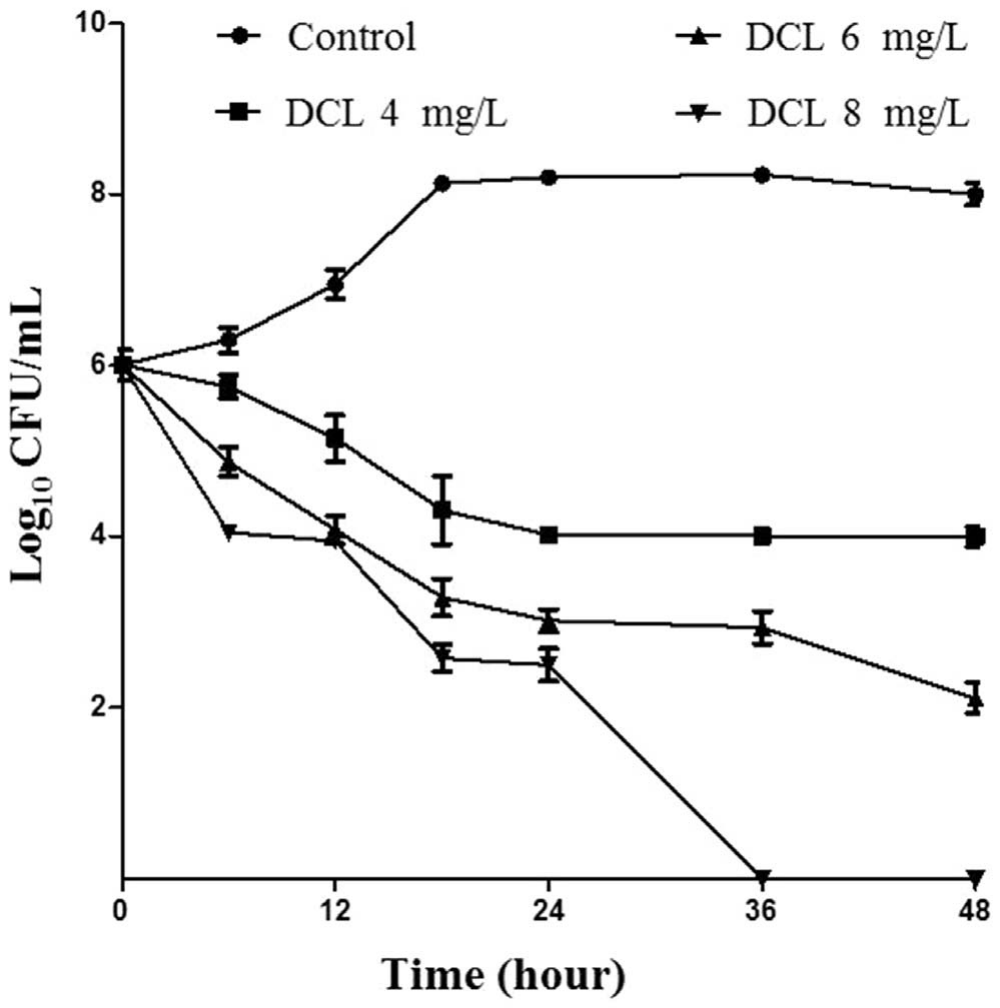
Time-course bactericidal activity. *H. pylori* ATCC43504 was cultured in Brucella broth without or with dehydrocostus lactone (DCL) at 1 (4 mg/L), 1.5 (6 mg/L), and 2 times (8 mg/L) the minimal inhibitory concentration. Viable count of *H. pylori* ATCC43504 was reduced in a concentration- and time-dependant manner. The bacterial strain survived for 48 h in 4 and 6 mg/L of DCL but died at 36 h in 8 mg/L of DCL. The mean values (± SD) for the log number of colony-forming unit (CFU)/mL were plotted.

Time course of bactericidal activity of DCL at different concentrations toward *H. pylori* ATCC43504 was likewise examined (Figure 2). The results revealed that viable count of the organism was reduced in a concentration- and time-dependant manner. The bacterial strain survived for 48 h in 4 and 6 mg/L of DCL but died at 36 h in 8 mg/L of DCL.

### Effect on the Viability of *H. pylori* at Varying pH Values

**Figure 3:**
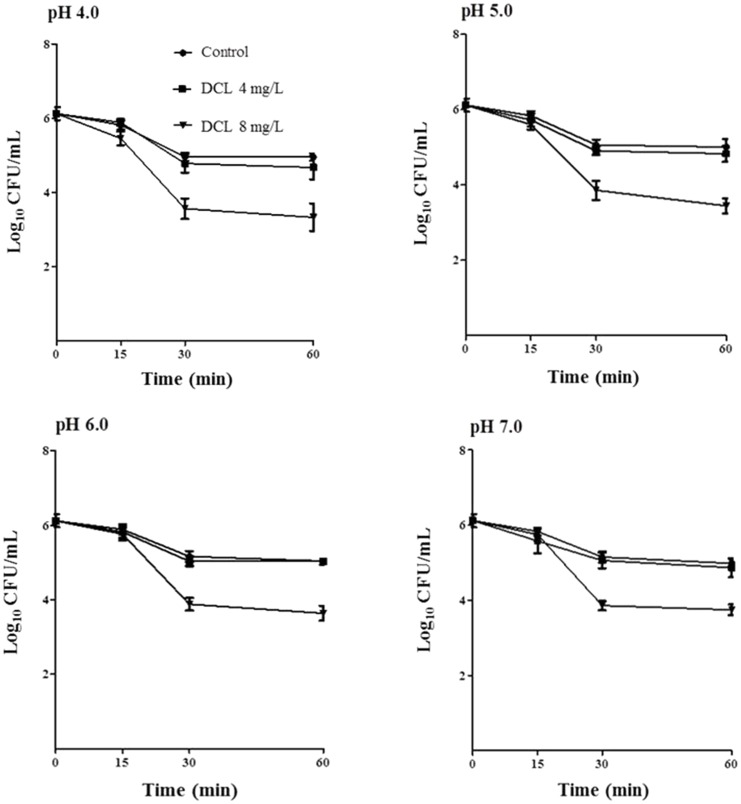
The bactericidal activity at various pH values. *H. pylori* ATCC43504 was cultured in Brucella broth without or with dehydrocostus lactone (DCL) at 1 (4 mg/L) and 2 times (8 mg/L) the minimal inhibitory concentration. DCL exhibited concentration-dependent bactericidal effects at all pHs (pHs 4.0, 5.0, 6.0, and 7.0). The activity of DCL did not differ significantly at the pH values examined. The mean values (± SD) for the log number of colony-forming unit (CFU)/mL were plotted.

The bactericidal activity of DCL toward *H. pylori* ATCC43504 at various pH values (4.0–7.0) was investigated (Figure 3). DCL exhibited concentration-dependent bactericidal effects at all pHs. The activity of DCL did not differ significantly at the pH values examined.

### Effect on Morphology of *H. pylori*

**Figure 4:**
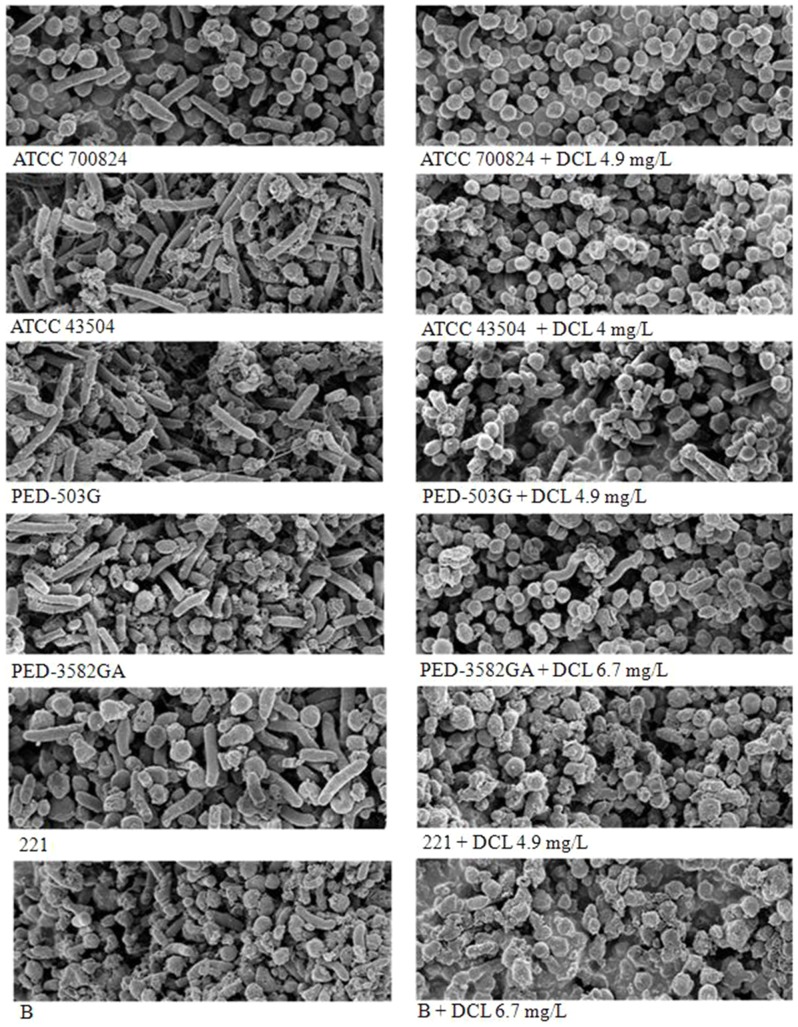
Scanning electron micrographs. The scanning electron micrographs for the *H. pylori* ATCC 700824 and five antibiotic-resistant strains of *H. pylori* treated without with dehydrocostus lactone (DCL) at the MIC showed conversion of the spiral to coccoid form, irrespective of antibiotic susceptibility of antibiotic-susceptible and -resistant strains. The coccoid-shaped cells were clustered and stuck to each other. Strains PED 503G, PED 3582GA, 221, *H. pylori* ATCC 43504, and B were resistant to amoxicillin, clarithromycin and tetracycline, metronidazole, metronidazole, and tetracycline and amoxicillin, respectively. Scale bar, 1 µm.

The morphostructural effects of the DCL treatment on the *H. pylori* ATCC 700824 and five antibiotic-resistant strains (*H. pylori* ATCC 43504, PED503G, PED 3582GA, 221, and B) of *H. pylori* were investigated (Figure 4). The scanning electron micrographs for the treated strain showed conversion of the spiral to coccoid form, irrespective of antibiotic susceptibility of antibiotic-susceptible and -resistant strains. The coccoid-shaped cells were clustered and stuck to each other.

**Figure 5:**
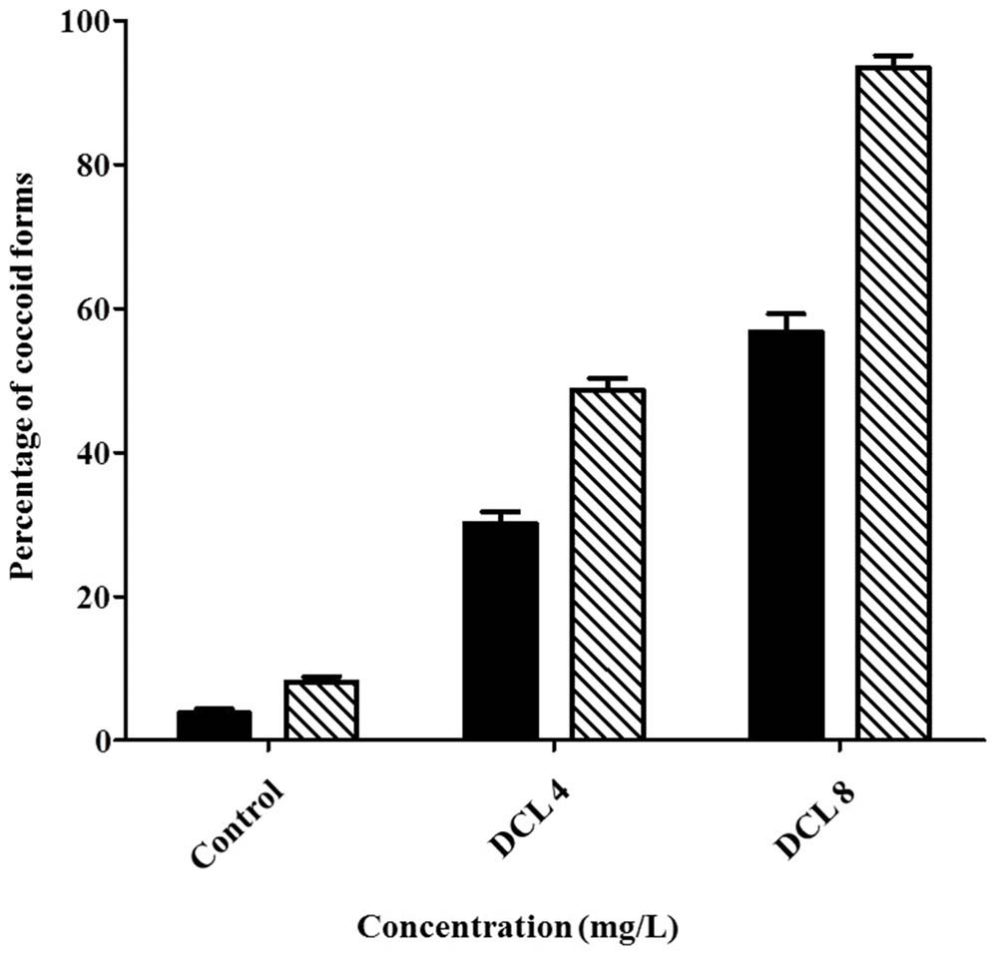
The proportion of coccoid versus spiral form. *H. pylori* ATCC43504 was cultured in Brucella broth without or with dehydrocostus lactone (DCL) at 1 (4 mg/L) and 2 times (8 mg/L) the minimal inhibitory concentration for 24 (▪) and 48 h (□). Effect of concentration (*F* = 1363.16; df  = 2, 15; *P*<0.0001) and exposure time (*F* = 188.66; df  = 1, 15; *P*<0.0001) on conversion of *H. pylori* to the coccoid form was significant when *H. pylori* controls were compared to DCL-treared *H. pylori*. The concentration by exposure interaction was also significant (*F* = 40.41; df  = 2, 15; *P*<0.0001). DCL caused considerable conversion to the coccoid form (58 versus 30% at 8 and 4 mg/L DCL for 24 h; 94 versus 49% at 8 and 4 mg/L DCL for 48 h).

The proportion of coccoid versus spiral form of *H. pylori* ATCC 43504 was determined at 4 and 8 mg/L of DCL for 24 and 48 h (Figure 5). Effect of concentration (*F* = 1363.16; df  = 2, 15; *P*<0.0001) and exposure time (*F* = 188.66; df  = 1, 15; *P*<0.0001) on conversion of *H. pylori* to the coccoid form was significant when *H. pylori* controls were compared to DCL-treared *H. pylori*. The concentration by exposure interaction was also significant (*F* = 40.41; df  = 2, 15; *P*<0.0001). DCL caused considerable conversion to the coccoid form (58 versus 30% at 8 and 4 mg/L of DCL for 24 h; 94 versus 49% at 8 and 4 mg/L of DCL for 48 h).

### Urease Inhibitory Activity

**Figure 6:**
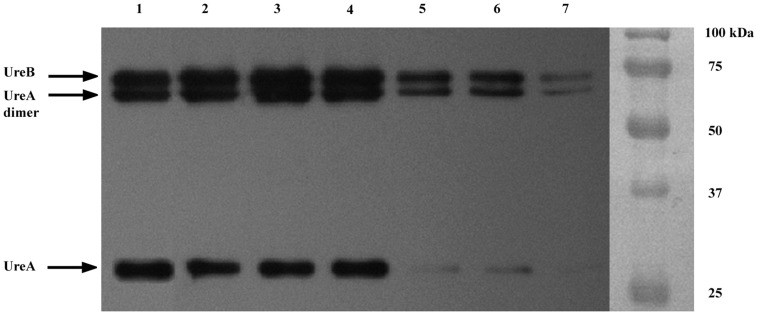
Western blot profiles. *H. pylori* ATCC 43504 urease was treated with dehydrocostus lactone (DCL) and (–)-epigallocatechin gallate (EGCG). The UreA (monomer) and UreB (dimer) bands were confirmed by Western blot with antibodies toward *H. pylori* urease. The UreB band and UreA dimer and monomer bands were not disappeared at 10 mM DCL. However, the UreA monomer band completely disappeared at 0.1 mM EGCG, and the UreA dimer and UreB bands were faint at the same concentration of the compound. Lanes: 1, ATCC 43504 urease; 2, 2 mM DCL; 3, 5 mM DCL; 4, 10 mM DCL; 5, 0.1 mM EGCG; 6, 0.2 mM EGCG; 7, 0.5 mM EGCG.

The *in vitro* urease inhibitory activity of DCL was compared with that of EGCG toward *H. pylori* ATCC 43504. Based on IC_50_ values, DCL showed no urease inhibition (IC_50_, >10 mM), whereas EGCG showed strong urease inhibition (IC_50_, 0.03 mM). Moreover, the UreA (monomer) and UreB (dimer) bands, as shown in Figure 6 (lane 1), were confirmed by Western blot with antibodies toward *H. pylori* urease. The UreB band and UreA dimer and monomer bands were not disappeared at 10 mM DCL. However, the UreA monomer band completely disappeared at 0.1 mM EGCG, and the UreA dimer and UreB bands were faint at the same concentration of the compound.

## Discussion

Various secondary substances, such as alkaloids, phenolics, and terpenoids, exist in plants, and jointly or independently they contribute to biological efficacy toward a variety of diseases [31]. Plant preparations are a potential for *H*. *pylori* therapy because some are selective and biodegrade to nontoxic products [32]. Anti-*H. pylori* constituents derived from plants include coumarins [33], flavonoids (e.g., (–)-epigallocatechin gallate, MIC 1–64 mg/L) [34], isothiocyanates (e.g., *D*-sulforaphane, MIC 0.125–8 mg/L) [35], phenolics [29], quinones (e.g., 2-(hydroxymethyl)anthraquinone, MIC 2 mg/L [36]; 2-methoxy-1,4-naphthoquinone (MIC, 0.156–0.625 mg/L for antibiotic-resistant strains) [37]), tannins (e.g., strictinin, MIC 6.25 mg/L; heteropylliin, MIC 12.5 mg/L) [38], terpenoids (e.g., 3-*O*-caffeoylbetulinic ascid, MIC 25 mg/L [38]; arjunglucoside I, MIC 1.9–7.8 mg/L for antibiotic-resistant strains) [39], and others [13], [37].

In the current study, the anti-*H. pylori* principle of *M*. *sieboldii* leaves was determined to be the guaianolide sesquiterpenoid dehydrocostus lactone. MIC of the constituent was between 4.0 and 6.7 mg/L toward two susceptibe *H. pylori* strains, although MIC of the natural compounds stated previously is between 1 and 100 mg/L. DCL was slightly less pronounced in the growth inhibitory and bactericidal activities than metronidazole, although the concentration of DCL was lower than that of either amoxicillin, clarithromycin, or tetracycline. The constituent was more active than the germacranolide sesquiterpenoids costunolide and parthenolide. Differences in the biological activites of different sesquiterpene lactones were reported to be because of differences in the number of alkylating structural elements, lipophilicity, molecular geometry, and the chemical environment of the target sulfhydryl group [40].

The pH is one of the factors affecting growth and antibiotic susceptibility of *H. pylori* [41]. The organism was reported to be frequently isolated from the gastric juice of patients at pHs below 3.0 [42]. In the current study, the bactericidal activity of DCL was not influenced by pH values examined, indicating that the antibacterial activity of DCL was not dependent on pH. Certain bactericidal agents toward *H. pylori* are dependent on pH. For example, EGCG has a potent bactericidal effect at pH 7 but not at pHs ≤5.0 [34]. The bactericidal activity of ecabet sodium, an antiulcer agent, increases under acidic conditions (pHs 4.0 and 5.0) [43], whereas the activity of antibiotics such as clarithromycin decreases under acidic conditions [44]. It has been suggested that ecabet sodium may bind to surface of cells and the level of binding is higher at low pH [43].

Conversion of the spiral to the coccoid form in *H. pylori* is caused by environmental factors or antibiotic treatment [1], [45]. The coccoid form has a lower level of metabolism and of protein and DNA synthesis than the spiral form [24]. It has been suggested that coccoid forms consist of the living bacteria and the dying bacteria [46], [47]. Unlike the former forms, the dying forms are not capable of recovering their virulence and causing the occurrence of diseases [46], [47]. In the current study, a proportional relationship between the coccoid number induced by DCL and its bactericidal property was observed. This suggested that most of the coccoid forms induced by DCL were the morphologic manifestation of bacterial cell death.

Investigations on the modes of action and the resistance mechanisms of natural antimicrobials may also contribute to the development of selective *H. pylori* therapeutic alternatives with novel target sites and future resistance management. The modes of action of plant secondary metabolites such as alkaloids, phenolics, and terpenoids have been well described by Wink [31]. *H. pylori* urease functions as both a colonization factor and a virulence factor because of the production of ammonia, which may contribute to the development of gastritis and peptic ulceration [1]. In addition, the prevalence and severity of antibiotic-resistant strains of *H*. *pylori* are increasing, and the development of safe and effective nonantibiotic agents is urgent for global public health. Certain phytochemicals such as capsaicin [48], isothiocyanates [35], 2-methoxy-1,4-naphthoquinone and spinasterol [37], and 1,2,3,4,6-penta-*O*-galloyl-β-D-glucopyranose and paeonol [13] are highly effective toward drug-resistant strains of *H. pylori* and are likely to be useful in resistance management strategies. The modes of action and the resistance mechanisms of anti-*H. pylori* drugs have been well reviewed by Gerrits et al. [6] and Francesco et al. [7].

In the current study, DCL exhibited potent growth inhibitory and bactericidal activity toward five strains resistant to amoxicillin (strains PED 503G and B), clarithromycin (strain PED 3582GA), metronidazole (starins 221 and *H. pylori* ATCC 43504), or tetracycline (strains PED 3582GA and B). This finding that DCL is virtually equal in antibacterial activity toward both antibiotic-susceptible and -resistant strains of *H. pylori* suggests that DCL and the penicillin amoxicillin, the macrolide clarithromycin, the nitroimidazole metronidazole, or the polyketide tetracycline do not share a common mode of action or elicit cross-resistance. This original finding indicates that materials derived from *M*. *sieboldii* leaves may hold promise for the development of novel and effective antibacterial products even toward currently antibiotic-resistant *H. pylori*. In addition, DCL did not inhibit urease which may indicate a different mode of action. Unlike EGCG, six 32 kDa (UreA) and six 66 kDa (UreB) subunits of the urease enzyme described previously [27] were not affected by DCL treatment. Detailed tests are needed to fully understand the exact antibacterial mode of action of DCL.

## Conclusions

*M*. *sieboldii* leaf-derived preparations containing dehydrocostus lactone could be useful as sources of antibacterial products for prevention or eradication of diseases caused by *H*. *pylori* in the light of their activity toward antibiotic-resistant *H. pylori* strains. The anti-*H*. *pylori* action of DCL may be an indication of at least one of the pharmacological actions of *M*. *sieboldii*. For the practical use of *M*. *sieboldii* leaf-derived preparations as novel anti-*H*. *pylori* products to proceed, further research is needed to establish their human safety and whether this activity is exerted *in vivo* after consumption of *M*. *sieboldii* leaf-derived products by humans. Dehydrocostus lactone has no acute oral toxicity on rat at 500 mg/kg [49]. Lastly, detailed tests are needed to understand how to improve anti-*H*. *pylori* potency (e.g. in combination with other antimicrobials (Gerrits et al. [6]) and stability for eventual commercial development.
